# Dual oncogenic roles of TPD52 and TPD52L2 in gastric cancer progression *via* PI3K/AKT activation and immunosuppressive microenvironment remodeling

**DOI:** 10.1093/bfgp/elaf015

**Published:** 2025-09-19

**Authors:** Hailong Li, Xiaqing Gao, Shuangming Guo, Shenfei Gao, Chunting Yang, Rong Su, Zhe Jing, Shuping Qiu, Ping Tang, Jing Han

**Affiliations:** The First Clinical Medical College, Gansu University of Chinese Medicine, No. 35 Dingxi East Road, Chengguan District, Lanzhou City, Gansu Province, Lanzhou 730000, P. R. China; Department of Scientific & Research, Affiliated Hospital of Gansu University of Chinese Medicine, No. 732 Jiayuguan West Road, Chengguan District, Lanzhou City, Lanzhou 730000, P. R. China; Department of General Practice, Luohu Clinical Institute of Shantou University Medical College, No. 47 Youyi Road, Luohu District, Shenzhen, Guangdong Province, Shenzhen 518000, P. R. China; The First Clinical Medical College, Gansu University of Chinese Medicine, No. 35 Dingxi East Road, Chengguan District, Lanzhou City, Gansu Province, Lanzhou 730000, P. R. China; Key Laboratory of Gansu Provincial Prescription Mining and Innovative Translational Laboratory, Gansu University of Chinese Medicine, No. 35 Dingxi East Road, Chengguan District, Lanzhou City, Gansu Province, Lanzhou 730000, P. R. China; The First Clinical Medical College, Gansu University of Chinese Medicine, No. 35 Dingxi East Road, Chengguan District, Lanzhou City, Gansu Province, Lanzhou 730000, P. R. China; Department of Gastroenterology, Xihua County People’s Hospital, No. 15 Jicheng Road, Chengguan Town, Xihua County, Zhoukou City, Hennan Province, Zhoukou 466600, P. R. China; The First Clinical Medical College, Gansu University of Chinese Medicine, No. 35 Dingxi East Road, Chengguan District, Lanzhou City, Gansu Province, Lanzhou 730000, P. R. China; The First Clinical Medical College, Gansu University of Chinese Medicine, No. 35 Dingxi East Road, Chengguan District, Lanzhou City, Gansu Province, Lanzhou 730000, P. R. China; The First Clinical Medical College, Gansu University of Chinese Medicine, No. 35 Dingxi East Road, Chengguan District, Lanzhou City, Gansu Province, Lanzhou 730000, P. R. China; The First Clinical Medical College, Gansu University of Chinese Medicine, No. 35 Dingxi East Road, Chengguan District, Lanzhou City, Gansu Province, Lanzhou 730000, P. R. China; Department of General Practice, Luohu Clinical Institute of Shantou University Medical College, No. 47 Youyi Road, Luohu District, Shenzhen, Guangdong Province, Shenzhen 518000, P. R. China; Department of Scientific & Research, Affiliated Hospital of Gansu University of Chinese Medicine, No. 732 Jiayuguan West Road, Chengguan District, Lanzhou City, Lanzhou 730000, P. R. China

**Keywords:** TPD52, TPD52L2, gastric cancer, immune cell infiltration

## Abstract

**Aim:**

TPD52 (tumor protein D52) and TPD52L2 (tumor protein D52-like 2), members of the TPD52 gene family, have been implicated in multiple malignancies. However, their roles in gastric cancer (GC) remain elusive. Herein, we integrated multiomics analyses and experimental validation to elucidate their prognostic and functional significance in GC.

**Methods:**

Utilizing The Cancer Genome Atlas (TCGA), Gene Expression Omnibus (GEO), and tissue microarray datasets, we analyzed TPD52/TPD52L2 expression patterns in patients with GC. Survival analysis, Cox regression, and nomogram construction were performed to assess prognostic value. Gene Ontology and Kyoto Encyclopedia of Genes and Genomes functional enrichment analysis and immune infiltration evaluation (Cell-type Identification By Estimating Relative Subsets Of RNA Transcripts‌/Estimation of STromal and Immune cells in MAlignant Tumour tissues using Expression data) (CIBERSORTx/ESTIMATE) were conducted to explore the molecular mechanisms involved. *In vitro* experiments (cell proliferation, migration, invasion, and apoptosis assays) were performed *via* lentivirus-mediated gene knockdown in gastric cancer cell lines AGS and MKN45 cells.

**Results:**

TPD52 and TPD52L2 were significantly overexpressed in GC tissues compared with their normal counterparts. Elevated TPD52L2 expression was significantly associated with advanced Tumor, Node, Metastasis (TNM) stage and independently predicted reduced overall survival according to multivariate Cox regression. Multivariate analysis identified TPD52L2 as an independent prognostic factor. Diagnostic Receiver Operating Characteristic (ROC) curves yielded area under the curve values of 0.813 (TPD52) and 0.807 (TPD52L2). The results of functional experiments suggested that TPD52/TPD52L2 knockdown inhibited proliferation, migration, G0/G1 arrest, and induced apoptosis. Mechanistically, TPD52/TPD52L2 silencing suppressed PI3K/Akt serine/threonine kinase (AKT)/mammalian target of rapamycin (mTOR) signaling and epithelial–mesenchymal transition marker expression.

**Conclusion:**

TPD52 and TPD52L2 are promising prognostic biomarkers in GC, with TPD52L2 exhibiting greater clinical relevance. Targeting these proteins may disrupt oncogenic signaling pathways and enhance immunotherapy efficacy, warranting further investigation in clinical trials.

## Graphical Abstract

**Graphical Abstract:**
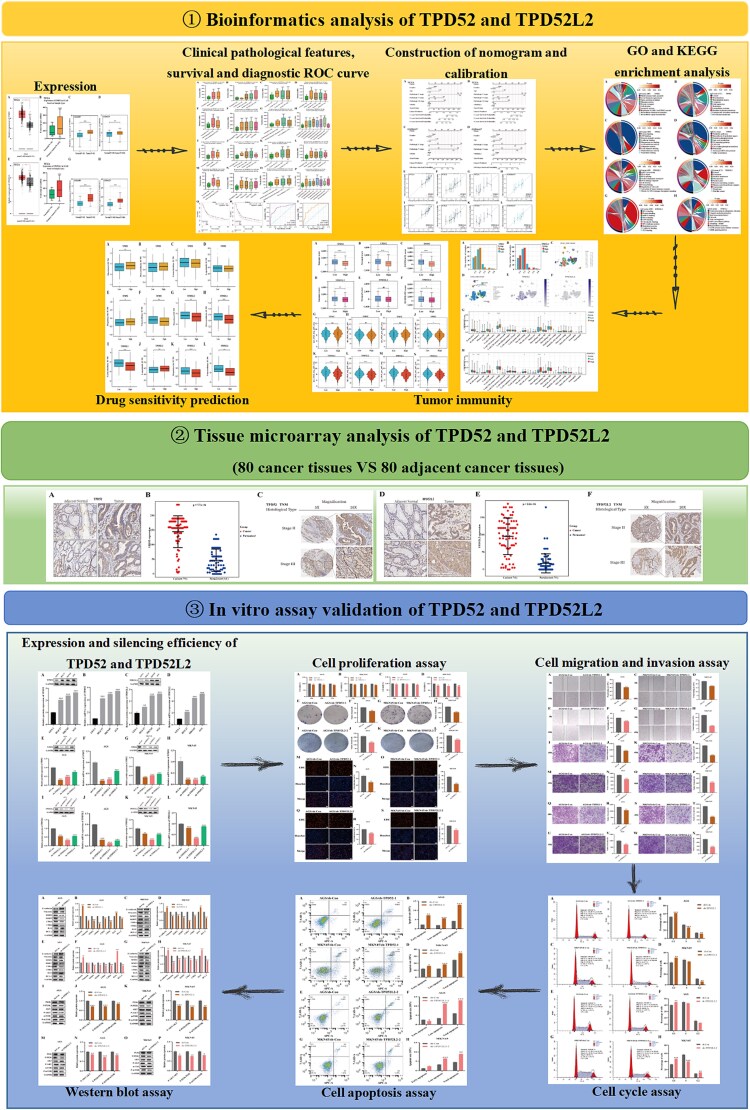

## Introduction

Gastric cancer (GC) is among the most prevalent malignant gastrointestinal tumors worldwide. According to the International Agency for Research on Cancer’s most recent estimates, there would be ~970 000 new cases of GC and 660 000 deaths in 2022, ranking sixth in the world in terms of morbidity and mortality [1]. According to data issued by the National Cancer Center in 2022, the incidence and mortality rates of GC in males are approximately twice those in women [2]. Established risk factors for gastric carcinogenesis include hereditary predispositions, dietary patterns, tobacco use, and *Helicobacter pylori* colonization [3–5]. Current clinical management employs stratified therapeutic paradigms: resectable tumors receive radical resection with perioperative chemotherapy, whereas advanced/metastatic cases necessitate personalized regimens [6]. The insidious onset of GC contributes to dismal 5-year survival rates (5%–20%) [7], although mortality reductions in high-incidence regions such as East Asia demonstrate the efficacy of endoscopic surveillance programs in enabling early intervention [8–10]. These findings demonstrate the importance of early diagnosis in guaranteeing appropriate therapy and improving patient survival rates in patients with GC. While endoscopy and biopsy are the most commonly used methods, they are often invasive and may not always be feasible for early-stage detection. Consequently, it is undeniably critical to develop novel and less invasive early detection technologies to reduce the prevalence of GC [6].

The transcripts of the tumor protein D52 family genes possess a typical coiled-coil motif structure with ~50 amino acid residues, which is a short hydrophilic polypeptide sequence with ~180–200 amino acid residues. The N-terminal and C-terminal PEST regions may play a role in protein stability [11]. The ability of tumor protein D52 family transcripts to be alternatively spliced is a significant indicator. As a result, distinct tumor protein D52 family genes can generate a wide variety of splicing variants. Specifically, TPD52 (hD52, D52, N8, N8L, PrLZ, PC-1, CRHSP-28, CSPP28), TPD52L1 (tumor protein D52-like 1) (hD53, D53, TPD53, hD53L1), TPD52L2 (hD54, D54, TPD54), and TPD52L3 (tumor protein D52-like 3) (hD55, D55, TPD55, NYD-SP28) are the four members of the tumor protein D52 family in mammals [12, 13]. However, TPD52, an oncogene in this gene family, is strongly expressed in ovarian cancer [14], colorectal cancer [15], pancreatic cancer [16], and other malignancies but is expressed at low levels in hepatocellular carcinoma [17] and leiomyosarcoma [18]. Additionally, TPD52L1 is significantly overexpressed in lymph node–positive breast cancer patients compared with that in lymph node–negative patients [19]. Indeed, TPD52L2 is highly expressed in lung adenocarcinoma [20], oral squamous cell carcinoma [21], and prostate cancer [22]. All these studies consistently reported elevated TPD52L2 levels. Notably, TPD52L3 presents a unique pattern: it is restricted to expression in healthy testicles, suggesting a potential role in spermatogenesis and testicular development [23]. Collectively, these findings establish the critical role of the D52 family in tumorigenesis.

The TPD52 family exhibits context-dependent oncogenicity: TPD52 drives cell cycle progression *via* CDK4/6 activation [24–26] while orchestrating oncogenesis through proliferation/apoptosis modulation and therapy resistance [16, 27, 28]. Paradoxically, in malignant insulinomas, TPD52 downregulation correlates with aggressive phenotypes and poor survival, serving as a tumor suppressor and a prognostic stratifier [29]. Conversely, TPD52L2 overexpression in lung adenocarcinoma is associated with TP53 mutations and immunosuppressive profiles, indicating its potential as a therapeutic biomarker [20]. Collectively, these findings position TPD52/TPD52L2 as pancancer therapeutic vulnerabilities. Furthermore, their functional characterization provides dual insights into tumor pathogenesis and precision oncology development. Nevertheless, their GC-specific regulatory networks, clinical associations, and druggable mechanisms warrant systematic exploration.

## Materials and methods

### Expression and prognostic analysis of TPD52 and TPD52L2 in gastric cancer

The Gene Expression Profiling Interactive Analysis (GEPIA) platform (http://gepia.cancer-pku.cn/) [30] serves as an interactive web server for analyzing tumor and normal tissue RNA-seq data from the TCGA and Genotype-Tissue Expression (GTEx) projects. Similarly, University of ALabama at Birmingham CANcer data analysis Portal (UALCAN) (https://ualcan.path.uab.edu/index.html) [31] provides a comprehensive platform for cancer Genomics and Proteomics (OMICS) data exploration. By leveraging these two widely used bioinformatics resources, we systematically examined TPD52 and TPD52L2 expression patterns in GC versus normal tissues and their correlations with clinicopathological parameters. Three GC datasets (GSE63089, GSE66229, and GSE84437) were retrieved from the GEO database (https://www.ncbi.nlm.nih.gov/geo). Additionally, survival analysis was conducted *via* the Kaplan–Meier plotter (https://www.xiantaozi.com/) [32], which evaluates gene expression–survival relationships across 21 tumor types using >35 000 samples. Moreover, to assess the diagnostic utility of TPD52 and TPD52L2 in GC, we performed ROC curve analysis on the TCGA-STAD cohort *via* the Xiantao platform (https://www.xiantaozi.com/), which enables quantitative evaluation of biomarker performance.

### Cox risk regression analysis and construction of a prognostic nomogram and calibration

Specifically, variables including sex, age, T stage, N stage, M stage, and TPD52 and TPD52L2 expression in patients with GC were included in univariate and multivariate Cox risk regression analyses *via* the GEO dataset and Xiantao database, respectively. To ensure robust statistical validation, the survival (3.3.1) software package was first utilized to test the proportional hazards hypothesis and perform Cox regression analysis. Furthermore, the rms (6.3--0) software package was employed to construct the nomogram correlation model and perform calibration analysis, completing the comprehensive prognostic assessment.

### Gene Ontology and Kyoto Encyclopedia of Genes and Genomes enrichment analysis

Single-gene correlations between TPD52 and TPD52L2 were initially screened *via* the Xiantao database. Specifically, TPD52-related genes were screened for | Cor| > 0.35 and *P* < .05 *via* the Pearson correlation coefficient criterion, whereas TPD52L2-related genes were screened for | Cor| > 0.50 and *P* < .05. The DAVID Functional Annotation Bioinformatics Microarray Analysis (DAVID DAVID Functional Annotation Bioinformatics Microarray Analysis (DAVID6.8)) database, which was subsequently used for annotation, visualization, and integrated discovery (https://david.ncifcrf.gov/) [33], offers a comprehensive suite of functional annotation tools for comprehending the biological significance underlying numerous genes. Eventually, the genes associated with TPD52 and TPD52L2 were entered into the DAVID database, and “*Homo sapiens*” was chosen for Gene Ontology (GO) and Kyoto Encyclopedia of Genes and Genomes (KEGG) enrichment analysis.

### Immune cell infiltration and immunotherapy analysis

The immune subtypes of GC patients were first obtained from University of Cingifornia Sisha Cruz (UCSC) Xena (http://xena.ucsc.edu/) [34], with subsequent investigations focusing on the distribution of these subtypes across different expression groups of TPD52 and TPD52L2. By leveraging the TISCH2 (Tumor Immune Single-cell Hub 2) platform (http://tisch.comp-genomics.org/) [35]—which provides comprehensive annotations of cell types at the single-cell level—researchers can more effectively characterize the tumor microenvironment (TME) across diverse tumor categories. Specifically, single-cell immune profiling *via* TISCH2 (http://tisch.comp-genomics.org/) revealed TPD52/TPD52L2-associated molecular networks. Additionally, the CIBERSORTx algorithm (https://cibersortx.stanford.edu/), a deconvolution-based tool, was used to quantify 22 immune cell subsets in GC tissues *via* RNA-seq data. Concurrently, the ESTIMATE algorithm (https://bioinformatics.mdanderson.org/estimate/) was applied to calculate key metrics of the TME, including tumor purity, stromal content, and immune infiltration scores, which were then stratified according to TPD52/TPD52L2 expression levels to evaluate their associations with immune microenvironment characteristics.

### Drug sensitivity prediction

The sensitivity of GC patients in the TCGA dataset to chemotherapy was specifically assessed in accordance with the *Genomics of Drug Sensitivity in Cancer (GDSC*) (https://www.cancerrxgene.org/) [37] database. To achieve this goal, the pRRophetic (4.0.3) software package was employed, which calculates half maximal inhibitory concentration (IC50) values to quantify drug sensitivity and further compares differences in the IC50 values of chemotherapy drugs across distinct expression groups of TPD52/TPD52L2.

### Human gastric cancer clinical specimens

Specifically, a tissue microarray (TMA) (**HStmA160CS01, Shanghai Outdo Biotech Company**) with the permit number SHYJS-CP-1701010 was approved by its ethics committee containing 160 points, comprising 80 GC tissues and 80 adjacent normal tissues, was analyzed. Additionally, the TMA analysis provided clinical diagnostic and demographic data, including sex, age, tumor size, grade, and T/N/M stage, to support downstream correlation analysis.

### Cell culture

For *in vitro* experiments, human gastric mucosal epithelial cells (GES-1) and human GC cell lines (HGC27, MKN45, AGS) were procured from Wuhan Zishan Biotechnology Co., Ltd. (Wuhan, China). The cells were cultured in medium supplemented with 10% fetal bovine serum (Servicebio, Cat No: G8002, Wuhan, China) and 1% penicillin–streptomycin (Servicebio, Cat No: G4003, Wuhan, China). The culture medium used was RPMI-1640 (Servicebio, Cat No: G4535, Wuhan, China), and the cells were maintained in a humidified incubator with 5% carbon dioxide at 37°C to ensure optimal growth conditions.

### Cell transfection

To investigate the functional roles of TPD52/TPD52L2, AGS, and MKN45 GC cells were seeded into 6-well plates at the optimal density. Upon reaching 70%–80% confluency, the cells were transduced with lentiviral vectors encoding TPD52/TPD52L2-targeting short hairpin RNA (shRNA)s (Shanghai Genechem Co., Ltd.) in the presence of a transduction enhancer (HitransG reagent). Following a 12–16-h incubation period to allow viral integration, the viral supernatant was replaced with fresh complete culture medium supplemented with 10% Fetal Bovine Serum (FBS). Stable transfectants were selected *via* puromycin dihydrochloride (Solarbio, Cat. No. P8230, Beijing, China) treatment for 14 consecutive days. Green fluorescent protein (GFP) GFP)-positive cells were visualized under an inverted fluorescence microscope (Olympus IX73, Tokyo, Japan) to confirm transduction efficiency. The expression levels of TPD52 and TPD52L2 in the infected cells were subsequently validated through western blot and RT–qPCR (quantitative reverse-transcription polymerase chain reaction) assays to ensure knockdown efficacy.

### Quantitative reverse-transcription polymerase chain reaction assay

Total RNA was extracted from cells *via* the MolPure® Cell/Tissue Total RNA Kit (Yeasen, Cat No: 19221ES50; Shanghai, China). Complementary DNA (cDNA) was synthesized using reverse transcription of Hifair® III 1st Strand cDNA Synthesis SuperMix for qPCR (gDNA digester plus) (Yeasen, Cat No: 11141ES60, Shanghai, China). Hieff® qPCR SYBR® Green Master Mix (No Rox) (Yeasen, Cat No: 11201ES08, Shanghai, China) was used for RT–qPCR amplification, and the manufacturer’s instructions were rigorously followed. After amplification, the fusion curve was evaluated, and the target gene’s relative expression level was determined *via* the 2^-ΔΔCt^ method. The primers used for the RT–qPCR experiment are listed in Table 1.

**Table 1:** Primers for the RT–qPCR assay.

Gene	Primer name	Sequences (5′to 3′)
TPD52	Forward	CAGAACATTGCCAAAGGG
	Reverse	CTGAGCCAACAGACGAAA
TPD52L2	Forward	TTCACAGGCAGGACAGAAGA
	Reverse	TTGAAGGTCGCAGAGTTCCT
GAPDH	Forward	CAGGAGGCATTGCTGATGAT
	Reverse	GAAGGCTGGGGCTCATTT

### Western blot assay

The collected cells were treated with Radio Immunoprecipitation Assay (RIPA) lysis buffer (Solarbio, Cat No: R0010, Beijing, China), Phenylmethanesulfonyl fluoride (PMSF) (Solarbio, Cat No: P0100, Beijing, China), or a protein phosphatase inhibitor (All-in-One, 100×) (Solarbio, Cat No: P1260, Beijing, China) for 30 min on ice. Following centrifugation, the supernatant was collected, and the protein content was measured *via* a bicinchoninic acid (BCA) protein quantification kit (Solarbio, Cat No: PC0020, Beijing, China). The supernatant was transferred to a 5× sodium dodecyl sulfate – polyacrylamide gel electrophoresis (SDS–PAGE) loading buffer solution containing β - mercaptoethanol/dithiothreitol (DTT) (Solarbio, Cat No: P1040, Beijing, China) and heat-denatured. The combination was kept at −20°C for later use. The SDS–PAGE gel was prepared on the basis of molecular weight, and the protein was transferred to a PVDF membrane (Merck, REF: IPVH00010, Darmstadt, Germany). The membrane was blocked at room temperature for 15 min with rapid sealing solution (NCM Biotech, Cat No: P30500, Suzhou, China) and then incubated overnight with a primary antibody at 4°C. The PVDF membranes were washed and incubated with the secondary antibody at room temperature for 1 h. The protein bands were detected with a Super Electrochemiluminescence (ECL) Detection Reagent Kit (Yeasen, Cat No: 36208ES60, Shanghai, China), and the gray values of the proteins were calculated with ImageJ software. The antibodies used in the experiment were as follows: TPD52 (Affinity, Cat No: DF14353, Jiangsu, China); TPD52L2 (GeneTex, Cat No: GTX88975, Irvine, CA, USA); glyceraldehyde-3-phosphate dehydrogenase (GAPDH) (Immunoway, Cat No: YM3029, Plano, USA); Horseradish Peroxidase (HRP)* goat anti-rabbit IgG (H + L) (Immunoway, Cat No: RS0002, Plano, USA); and HRP* goat anti-mouse IgG (H + L) (Immunoway, Cat No: RS0001, Plano, USA). HRP* polyclonal rabbit anti-goat IgG (H + l) (Immunoway, Cat No: RS030221, Plano, USA) was used.

### C‌CK8, colony formation, and 5-Ethynyl-2'-deoxyuridine (EdU) assays

First, the cells were seeded into 96-well plates at 5 × 10^3^ cells/well for the Cell Counting Kit-8 (CCK8) assay. CCK8 solution (NCM Biotech, Cat No: C6005, Suzhou, China) was used to detect cell viability at 24, 48, and 72 h, after which the cells were incubated at 37°C for 2–3 h. Finally, the absorbance value (optical density, OD) of the cells was measured at 450 nm.

To evaluate long-term proliferative capacity, cells were seeded into 6-well plates at a density of 1 × 10^3^ cells/well and cultured for 14 days to conduct a colony formation assay. ​After incubation, the cell colonies were fixed with 4% paraformaldehyde (Solarbio, Cat No: P1110, Beijing, China), stained with crystal violet staining solution (Servicebio, Cat No: G1014, Wuhan, China), and subsequently​ quantified *via* manual counting. For mechanistic analysis of DNA synthesis, an EdU assay was performed with cells seeded in 6-well plates. ​Specifically, proliferating cells were labeled *via* the BeyoClick™ EDU Kit (Alexa Fluor 555; Beyotime, China, Cat# C0075S) following the manufacturer’s protocol. ​After labeling, images were captured under an inverted fluorescence microscope (Nikon Eclipse Ti2), ​followed by quantification​ of EdU^+^ cells (red) and Hoechst^+^ nuclei (blue) *via* ImageJ. The proliferation rate was calculated as (EdU^+^ cells (red)/Hoechst^+^ cells) (blue) × 100%.

### Wound healing assay

The cells were seeded onto 6-well plates, and, when the density reached 90%, a 200 μl sterile pipette tip was used to create a wound surface. The residual cells were rinsed with Phosphate Buffer Saline (PBS), and 2 ml of PMI-1640 media containing 2% fetal bovine serum was added to culture the cells. The migration distances of the cells were measured at 0 and 48 h.

### Transwell assay

For ​Transwell migration assays, cells (5 × 10^4^ cells/well) were seeded ​in 8 μm pore Transwell chambers​ with serum-free medium, while the lower chamber contained 10% FBS-containing medium. After ​48 h of incubation at 37°C in 5% CO₂, the cells were fixed with 4% paraformaldehyde for 15 min and stained with crystal violet for 15 min ​in the dark, and ​nonmigrated cells were removed​ *via* a cotton swab. The migrated cells were ​air-dried at room temperature for 30 min​ and ​quantified under a microscope. ​For invasion assays, Matrigel-coated membranes (1:8 dilution, polymerized for 4 h at 37°C) were used, and the subsequent steps followed the same migration protocol.

### Cell cycle assay

A Cell Cycle Staining Kit (MULTI SCIENCES, Cat No: CCS012, Hangzhou, China) was used to evaluate the cell cycle according to the manufacturer’s instructions. ​First, the cells were digested with 0.25% trypsin digestion solution (Servicebio, Cat No: G4012, Wuhan, China) and collected in centrifuge tubes. The supernatant was centrifuged, and the samples were then washed once with PBS. ​Next, the cells were resuspended in 500 μl of 1× binding buffer, followed by the addition of 5 μl of Annexin V-APC and 10 μl of 7-AAD to each tube. Next, the cells were oscillated and incubated for 5 min at room temperature in the dark, and ​the samples were detected *via* flow cytometry.

### Apoptosis assay

Apoptosis was evaluated *via* an apoptosis staining kit (MULTI SCIENCES, Cat No: AP105, Hangzhou, China) in accordance with the manufacturer’s instructions. ​First, the cells were digested with a 0.25% trypsin solution and washed with precooled PBS. ​Second, the cells were resuspended in 500 μl of 1× binding buffer, followed by the addition of 5 μl of Annexin V-APC and 10 μl of 7-AAD to each tube. After vortexing, the cells were incubated at room temperature for 5 min and kept in the dark. ​Finally, the samples were detected *via* flow cytometry.

### Statistical analysis

The experimental results were statistically analyzed and plotted *via* GraphPad Prism 9.3 software. The Kaplan–Meier survival curve was analyzed *via* the log-rank test. Independent-samples *t*-tests or Wilcoxon tests were used for comparisons between two groups, and one-way ANOVA with Tukey’s *post hoc* test was used for comparisons among multiple groups, where *P* < .05 was considered statistically significant. For all high-throughput analyses (gene expression correlation, immune infiltration, and drug sensitivity), *P*-values were adjusted *via* Benjamini–Hochberg false discovery rate (FDR) correction. Significant thresholds were set at FDR-adjusted *P* < .05.

## Results

### Differential expression and prognosis of TPD52 and TPD52L2 in the datasets

**Figure 1:**
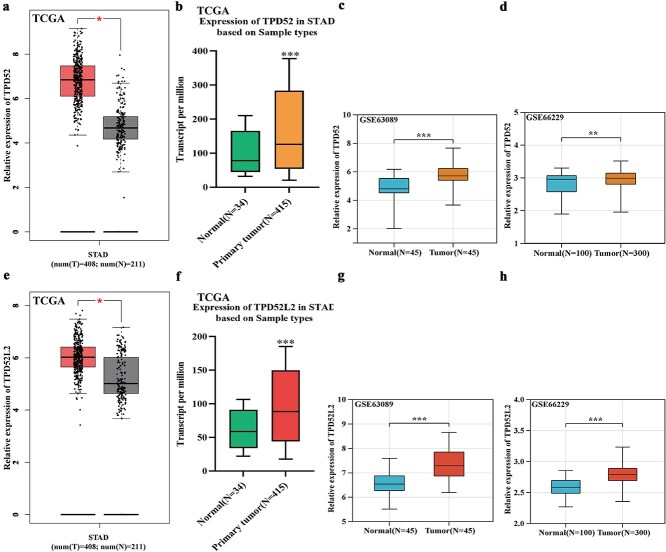
Differential expression of TPD52 and TPD52L2 in GC and normal tissues. (a, e) Expression levels of TPD52 and TPD52L2 in GC and normal tissues in the GEPIA database. (b, f) Expression levels of TPD52 and TPD52L2 in GC and normal tissues in the UALCAN database. (c, g) Expression levels of TPD52 and TPD52L2 in GC and normal tissues in the GSE63089 dataset. (d, h) Expression levels of TPD52 and TPD52L2 in GC and normal tissues in the GSE66229 dataset (compared with normal tissues, ^*^P < .05, ^**^P < .01, ^***^P < .001).

**Figure 2:**
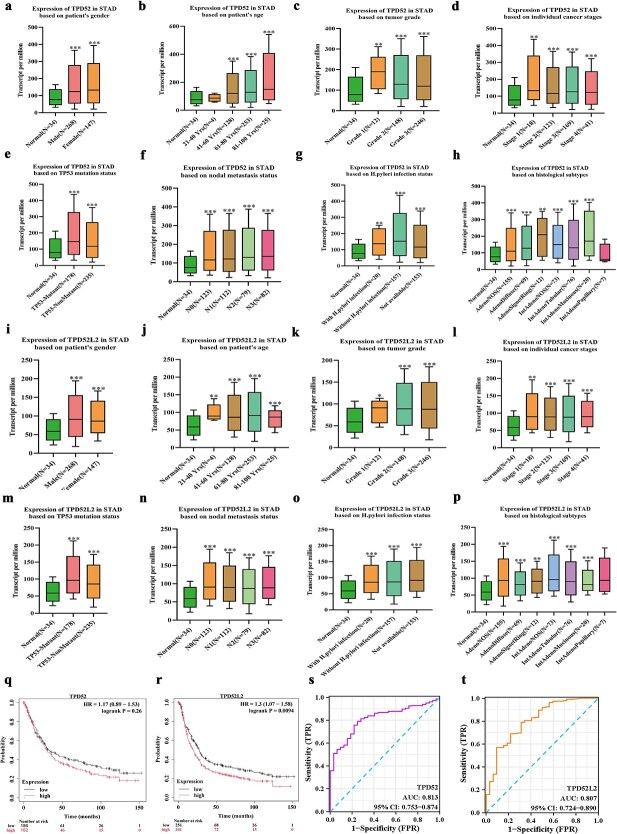
Clinicopathological features and prognosis of TPD52 and TPD52L2 expression in patients with GC. (a–h) Clinicopathological features and prognosis associated with TPD52 expression in patients with GC. (i–p) Clinicopathological features and prognosis associated with TPD52L2 expression in patients with GC. (Q-) KM PLOTTER survival time analysis of TPD52 in GC. (R) KM PLOTTER survival time analysis of TPD52L2 in GC. (s, t) Diagnostic ROC curve analysis of TPD52 in GC. (S-T) Diagnostic ROC curve analysis of TPD52L2 in GC (compared with normal tissues, ^*^P < .05, ^**^P < .01, ^***^P < .001).

Multiple database analysis (GEPIA/UALCAN) revealed significant upregulation of TPD52 and TPD52L2 in GC *versus* normal tissues (TCGA: Fig. 1a, b, e, and  f; GEO validation: Fig. 1c, d, g, and  h). Clinical significance analysis revealed elevated TPD52 expression in male, TP53-mutated, early-stage (grade 1/stage 1), and *H. pylori*-negative patients, with progressive increases observed with aging and lymph node metastasis (Fig. 2a–h). Similarly, TPD52L2 was more highly expressed in the female, TP53-mutated, and N0-stage subgroups (Fig. 2i–p). Kaplan-Meier (KM) plot survival time analysis revealed that the high TPD52L2 expression group had a shorter survival time (Fig. 2q and r), whereas there were no differences between the high TPD52 expression group and the low TPD52 expression group. Both genes suggested strong diagnostic potential (area under the curve [AUC]: TPD52 = 0.813, TPD52L2 = 0.807; Fig. 2s and t).

### Independent diagnostic value of TPD52 and TPD52L2 expression in gastric cancer

**Figure 3:**
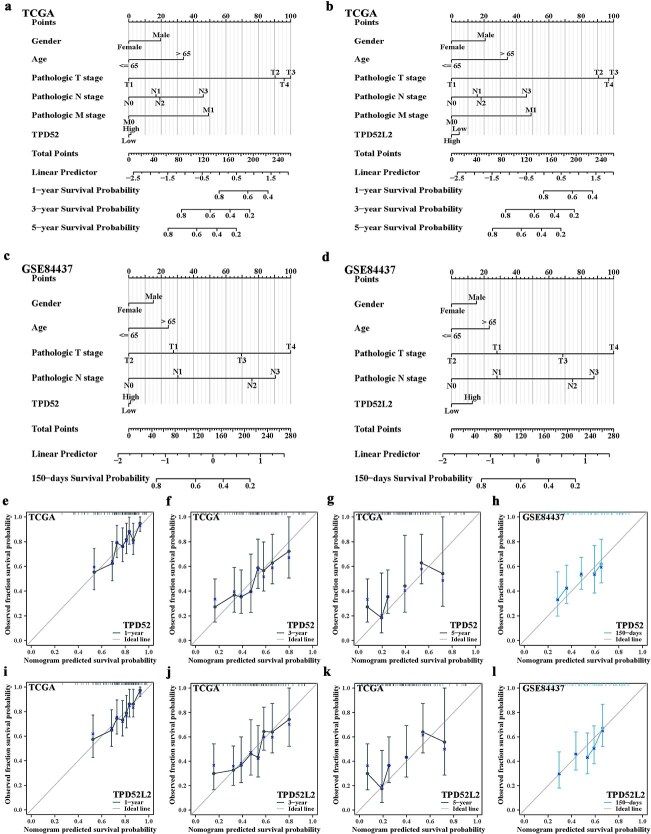
Construction of the nomogram and calibration in the TCGA and GSE84437 datasets. (a, b) Nomograms of clinicopathological features associated with TPD52 and TPD52L2 expression in the TCGA dataset. (c, d) Nomograms of clinicopathological features associated with TPD52 and TPD52L2 expression in the GSE84437 dataset. (e–l) Calibration of GC patient survival at 1 year, 3 years, 5 years, and 150 days in the TCGA and GSE84437 datasets.

We performed Cox regression analysis on the TCGA/GEO cohorts to assess prognostic factors (sex, age, TNM stage, and TPD52/TPD52L2 expression) in patients with GC. Univariate screening revealed that age > 65 years, T3/T4 stage, and nodal/metastatic involvement (N1/N3/M1) are poor prognostic indicators. Multivariate adjustment confirmed that age > 65, N3, and M1 were independent OS risk factors (Supplementary Tables S1 and S2), with the GSE84437 validation replicating age > 65, N2, and N3 associations (Supplementary Tables S3 and S4). A weighted nomogram integrating these covariates enabled quantitative survival probability prediction (Fig. 3a–d). The calibration plots suggested high 1/3-year predictive accuracy (Fig. 3e–l).

### Enrichment analysis of TPD52- and TPD52L2-related genes

**Figure 4:**
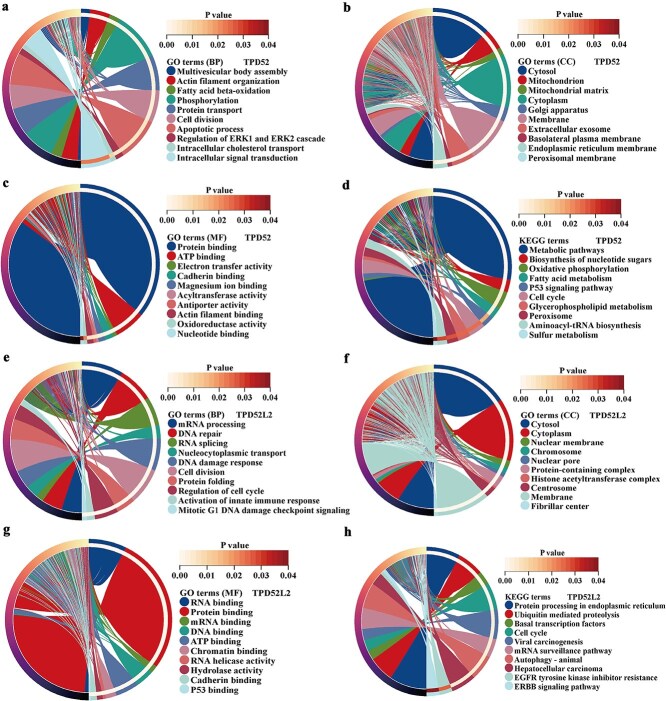
GO and KEGG enrichment analyses of TPD52- and TPD52L2-related genes. (a–c) GO enrichment analysis of TPD52-related genes. (d) KEGG enrichment analysis of TPD52-related genes. (e–g) GO enrichment analysis of TPD52L2-related genes. (h) KEGG enrichment analysis of TPD52L2-related genes.

Functional enrichment analysis of genes coexpressed with Xiantao-derived TPD52 and TPD52L2 revealed distinct tumor-related pathways. TPD52-linked genes showed GO enrichment in phosphorylation signaling (Biological Process, BP), mitochondrial/cytoplasmic localization (Cell Components, CC), and cadherin binding (molecular function, MF) (Fig. 4a–c), with KEGG mapping to metabolism and p53 pathways (Fig. 4d). Conversely, the TPD52L2 network suggested the involvement of the DNA damage response (BP), chromosomal organization (CC), and RNA binding motif (MF) (Fig. 4e–g), whereas the KEGG analysis suggested the involvement of the cell cycle deregulation and epidermal growth factor receptor (EGFR) resistance pathways (Fig. 4h).

### Immune infiltration analysis and analysis of the benefits of immunotherapy

**Figure 5:**
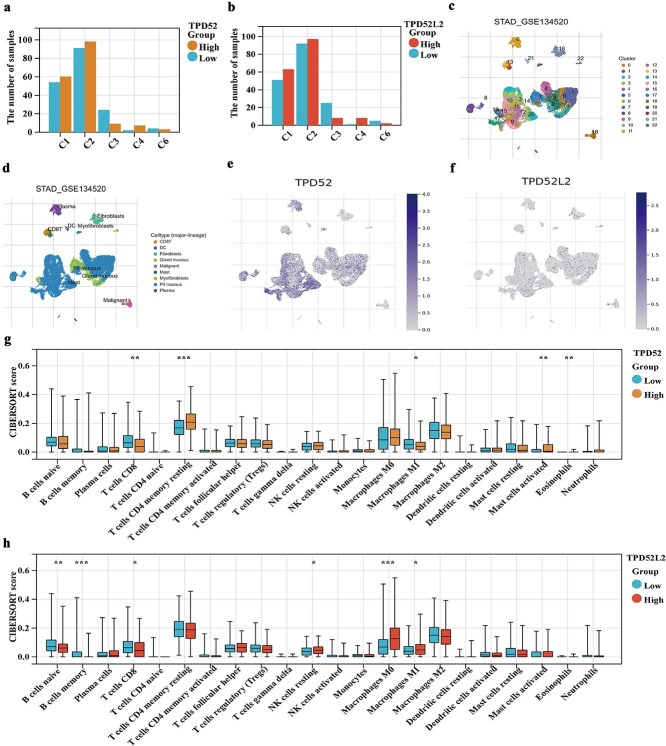
Distribution of immune subtypes and analysis of immune infiltration in different TPD52 and TPD52L2 expression groups. (a, b) Distribution of immune subtypes in different expression groups of TPD52 and TPD52L2. (c–f) The concentrations of TPD52 and TPD52L2 in TME cells were analyzed *via* single-cell analysis. (g, h) Infiltration levels of immune cells in different TPD52 and TPD52L2 expression groups. (compared with the TPD52 or TPD52L2 low-expression groups, ^*^P < .05, ^**^P < .01, ^***^P < .001).

The TCGA database is classified into six subgroups on the basis of tumor patients’ immunological status: wound healing (immunological C1), Interferon (IFN)-gamma dominant (immune C2), inflammatory (immune C3), lymphocyte depleted (immune C4), immunological quotient (immune C5), and TGF-beta dominant (immune C6). Compared with the low-TPD52 and TPD52L2 expression groups, the high-TPD52L2 expression group presented greater distributions of C1, C2, and C4 and lower distributions of C3 and C6. To investigate the distribution of prognostic genes in TME cells, TPD52 and TPD52L2 enrichment was assessed *via* single-cell analysis. TPD52 was highly concentrated in plasma, CD8+ T cells, mast cells, pit mucous, and gland mucous. TPD52L2 was predominantly found in pit and gland mucous (Fig. 5c–f). Next, immune cell infiltration was analyzed in patients in various TPD52 and TPD52L2 expression groups. Compared with the TPD52 low-expression group, the high-expression group presented higher levels of resting memory CD4 T cells, mast cell activation, and eosinophil infiltration but lower levels of infiltrating CD8 T cells (Fig. 5g). Compared with the TPD52L2 low-expression group, the high-expression group had higher infiltration levels of resting NK cells, M0 macrophages, and M1 macrophages but lower infiltration levels of naïve B cells, memory B cells, and CD8+ T cells (Fig. 5h).

**Figure 6:**
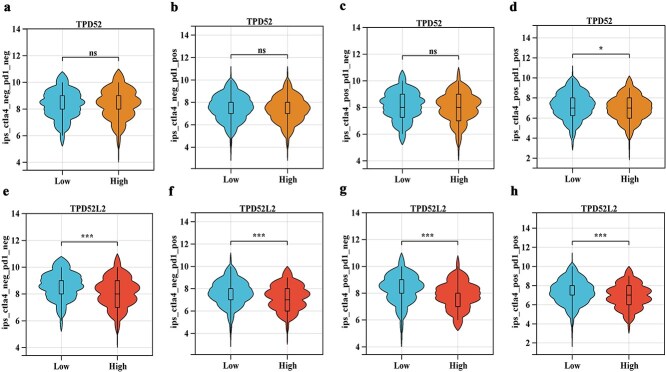
Bioinformatic prediction of the immunotherapy response of different expression groups of TPD52 and TPD52L2 to immunotherapy. (a–c) Analysis of immune, stromal, and ESTIMATE scores in different TPD52 expression groups. (d–f) Analysis of immune, stromal, and ESTIMATE scores in different TPD52L2 expression groups. (g–j) Analysis of IPS scores in different TPD52 expression groups. (k–n) Analysis of IPS scores in different TPD52L2 expression groups (compared with the TPD52 or TPD52L2 low-expression groups, ^*^P < .05, ^**^P < .01, ^***^P < .001, ns represents no statistical significance).

ESTIMATE database analysis revealed that the immune, stromal, and ESTIMATE scores of the TPD52 low-expression group were greater than those of the high-expression group (Fig. 6a–c). The immune and ESTIMATE scores were greater in the low-TPD52L2 expression group than in the high-TPD52L2 expression group, while the stromal score did not differ significantly between the two groups (Fig. 6d–f). CTLA4 and PD1 were analyzed immunophenoscore (IPS) and separated into four groups: ips_ctla4_neg_pd1_neg, ips_ctla4_neg_pd1_pos, ips_ctla4_pos_pd1_neg, and ips_ctla4_pos_pd1_pos. The higher the IPS is, the more immunogenic the patient is. As a result, the The Cancer Immunome Database (TCIA) database was used to examine the IPS scores of the various TPD52 and TPD52L2 expression groups. The results revealed that the ips_ctla4_pos_pd1_pos of the TPD52 low-expression group was greater than that of the high-expression group, and all four IPS score groups of the TPD52L2 low-expression group were higher than those of the high-expression group. Lower IPS scores in the high-expression groups ​suggest a potential correlation​ with reduced immunotherapy efficacy, ​although clinical response data are lacking. These findings suggested that immunotherapy was more effective in patients with reduced expression of TPD52 and TPD52L2 (Fig. 6g–n).

**Table 2:** Relationships between the expression of TPD52 in GC and clinicopathological parameters according to tissue microarray analysis.

	Variables	TPD52 expression		Total (*N*)	*P*-value
		Low	High		
Gender					.192
	Male	0	14	14	
	Female	10	51	61	
Age					.309
	≤64	3	34	37	
	>64	7	31	38	
Tumor_size					.471
	≤5 cm	4	38	42	
	>5 cm	5	24	29	
T					.478
	I-III	8	41	49	
	IV	2	24	26	
N					1
	N0	2	13	15	
	N1/N2/N3	8	52	60	
M					**.046**
	M0	7	61	68	
	M1	3	4	7	
Grade					.154
	II	4	44	48	
	III	6	21	27	
TNM					.723
	I-II	4	21	25	
	III-IV	6	44	50	

*Note:* P < 0.05, the expression of TPD52 is correlated with the M stage.

### Expression of TPD52 and TPD52L2 and sensitivity analysis of different drugs

**Figure 7:**
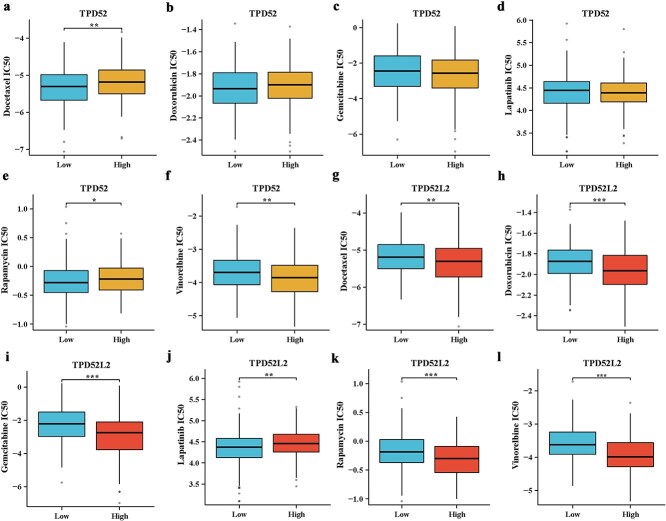
Drug sensitivity analysis of different expression groups of TPD52 and TPD52L2. (a–f) Sensitivity of different TPD52 expression groups to docetaxel, doxorubicin, gemcitabine, lapatinib, rapamycin, and vinorelbine. (g–l) Sensitivity of different TPD52L2 expression groups to docetaxel, doxorubicin, gemcitabine, lapatinib, rapamycin, and vinorelbine (compared with the TPD52 or TPD52L2 low-expression groups, ^*^P < .05, ^**^P < .01, ^***^P < .001).

Chemotherapy remains a cornerstone of GC treatment. To predict drug sensitivity, we calculated IC50 values for common chemotherapeutics on the basis of TCGA transcriptomic profiles. Tumors with low TPD52 expression exhibited an enhanced response to docetaxel and rapamycin but greater sensitivity to vinorelbine in patients with high TPD52 expression (Fig. 7a–f). Conversely, low TPD52L2 expression correlated with lapatinib responsiveness, whereas high expression was associated with broader drug sensitivity (docetaxel, doxorubicin, gemcitabine, rapamycin, and vinorelbine; Fig. 7g–l). Notably, the responses to doxorubicin, gemcitabine, and lapatinib were independent of TPD52 levels. These findings highlight the clinical potential of TPD52/TPD52L2 expression stratification for personalized chemotherapy in patients with GC.

### Expression of TPD52 and TPD52L2 and clinicopathological features

**Figure 8:**
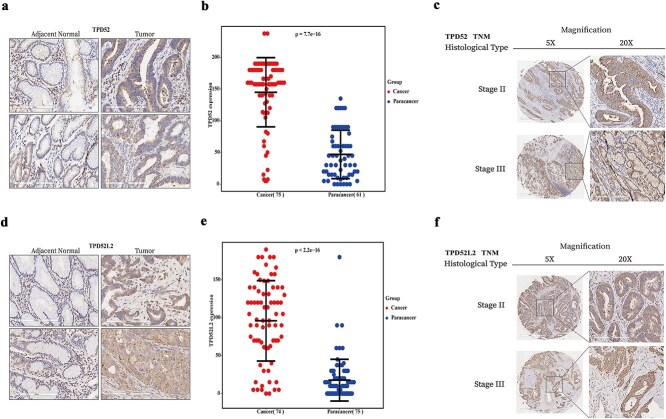
Tissue microarray analysis of TPD52 and TPD52L2 in GC and matched adjacent tissues. (a) Immunohistochemical staining of TPD52 in GC tissues and paracancerous tissues. (b) Expression levels of TPD52 in GC tissues and paracancerous tissues. (c) Immunohistochemical staining of TPD52 at stages II and III of the TNM stage. (d) Immunohistochemical staining of TPD52L2 in GC tissues and paracancerous tissues. (e) Expression levels of TPD52L2 in GC tissues and paracancerous tissues. (f) Immunohistochemical staining of TPD52L2 at stages II and III of the TNM stage.

Through meticulous examination of TPD52 and TPD52L2 protein expression in GC specimens, we determined their clinical significance. Immunohistochemistry revealed that the predominant cytoplasmic localization of both proteins in malignant cells was significantly elevated in tumors versus adjacent nontumor tissues (Fig. 8a and b for TPD52; *P* < .01, Fig. 8d and e for TPD52L2).

Notably, TPD52 expression was associated with distant metastasis (M stage, *P* = .003), whereas TPD52L2 expression was associated with advanced TNM stage (*P* = .011) (Tables 2 and 3). Immunohistochemical staining further revealed that TPD52 and TPD52L2 were expressed in the cytoplasm of GC tissues at stages II/III of TNM (Fig. 8c for TPD52; Fig. 8f for TPD52L2).

**Table 3:** Relationships between the expression of TPD52L2 in GC and clinicopathological parameters according to tissue microarray analysis.

	Variables	TPD52L2 expression		Total (*N*)	*P*-value
		Low	High		
Gender					1
	Male	13	1	14	
	Female	53	7	60	
Age					1
	≤64	33	4	37	
	>64	33	4	37	
Tumor_size					.252
	≤5 cm	39	3	42	
	>5 cm	23	5	28	
T					1
	I–III	43	5	48	
	IV	23	3	26	
N					.339
	N0	14	0	14	
	N1/N2/N3	52	8	60	
M					1
	M0	59	8	67	
	M1	7	0	7	
Grade					.247
	II	41	7	48	
	III	25	1	26	
TNM					**.048**
	I–II	24	0	24	
	III–IV	42	8	50	

*Note:* P < 0.05, the expression of TPD52L2 is correlated with the TNM stage (P < 0.05).

Multivariate analysis confirmed that neither protein correlated with sex, age, tumor size, T/N stage, nor histological grade (all *P* > .05). These findings implicate TPD52 and TPD52L2 as stage-specific biomarkers with prognostic potential in GC progression. ​These findings imply that TPD52 and TPD52L2 may serve as potential prognostic markers for patients with GC.

### Knockdown of TPD52 and TPD52L2 inhibited the proliferation of gastric cancer cells

**Figure 9:**
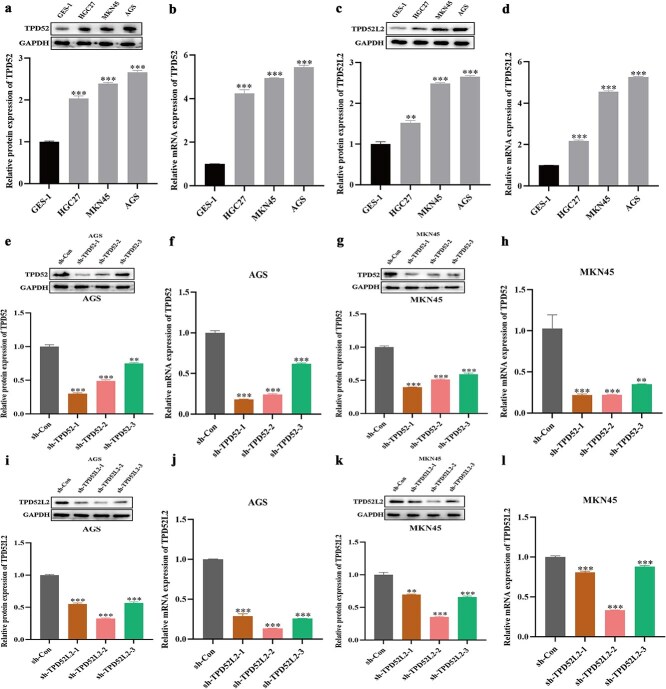
Expression of TPD52 and TPD52L2 in *GC* cell lines and knockdown efficiency in GC cells. (a–d) mRNA and protein expression levels of TPD52 and TPD52L2 in a *human gastric mucosal epithelial cell* line and gastric cancer cell lines. (e–h) RT–qPCR and western blot assays were used to verify the knockdown efficiency of TPD52 and TPD52L2 in AGS and MKN45 cells. (i–l) RT–qPCR and western blot assays were used to verify the knockdown efficiency of TPD52L2 and TPD52L2 in AGS and MKN45 cells. (compared with the GSE-1 or sh-Con groups, ^*^P < .05, ^**^P < .01, ^***^P < .001).

To investigate the roles of TPD52 and TPD52L2 in GC pathogenesis, we first analyzed their expression patterns in normal gastric mucosa cells (GES-1) versus GC cell lines (HGC27, MKN45, AGS).​​ qRT–PCR and immunoblotting revealed that TPD52 and TPD52L2 expression was significantly elevated in malignant cell lines compared with normal controls (Fig. 9a–d). To functionally validate these observations,​​we performed shRNA-mediated knockdown in MKN45 and AGS cells. Fluorescence microscopy confirmed ≥85% transfection efficiency (Supplementary Fig. S1), whereas subsequent qRT–PCR and immunoblotting verified successful target suppression. Among the screened constructs, ​sh-TPD52-1​ and ​sh-TPD52L2-2​ exhibited maximal knockdown efficacy (Fig. 9e–l) and were therefore selected for mechanistic studies.

**Figure 10:**
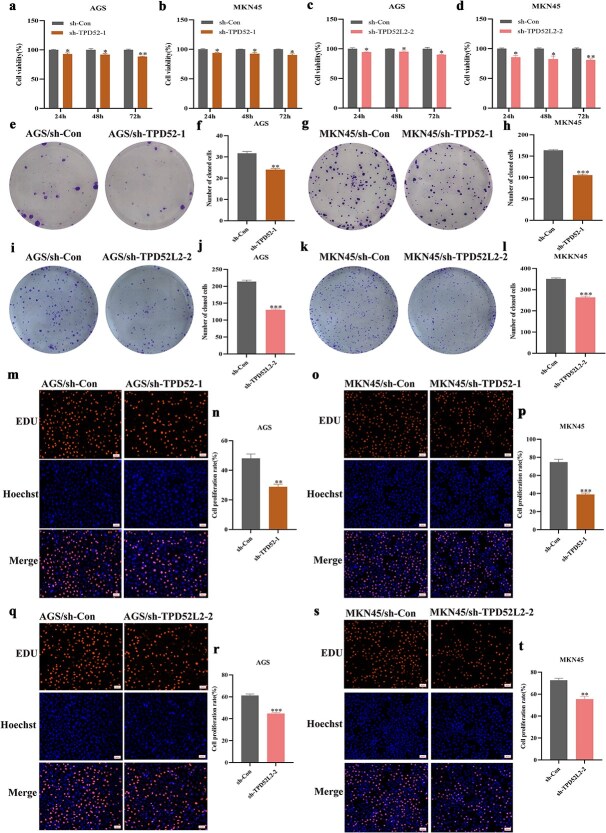
The influence of TPD52 and TPD52L2 knockdown on the proliferation of GC cells. (a–d) A CCK-8 assay was used to assess the viability of cells with silenced TPD52 and TPD52L2 expression. (e–l) A cell colony formation assay was conducted to evaluate the cloning efficiency of cells in which TPD52 and TPD52L2 had been silenced. (m–t) The EdU assay was utilized to measure the DNA synthesis capacity in cells with silenced TPD52 and TPD52L2. (compared with the sh-Con groups, ^*^P < .05, ^**^P < .01, ^***^P < .001).

Functional analyses revealed that TPD52/TPD52L2 knockdown significantly inhibited GC cell proliferation. Specifically, CCK-8 assays demonstrated reduced viability in the shRNA-transfected groups compared with the scramble-RNA control groups (Fig. 10a–d), the colony formation capacity​ was markedly suppressed (Fig. 10e–l), and the EdU incorporation rates​ decreased significantly (Fig. 10m–t) in the silenced groups compared with the control groups. Taken together, these collective results establish TPD52/TPD52L2 as critical regulators of GC cell proliferation *in vitro*, suggesting their potential as therapeutic targets for gastric cancer intervention​.

### Knockdown of TPD52 and TPD52L2 inhibited the metastasis of gastric cancer cells

**Figure 11:**
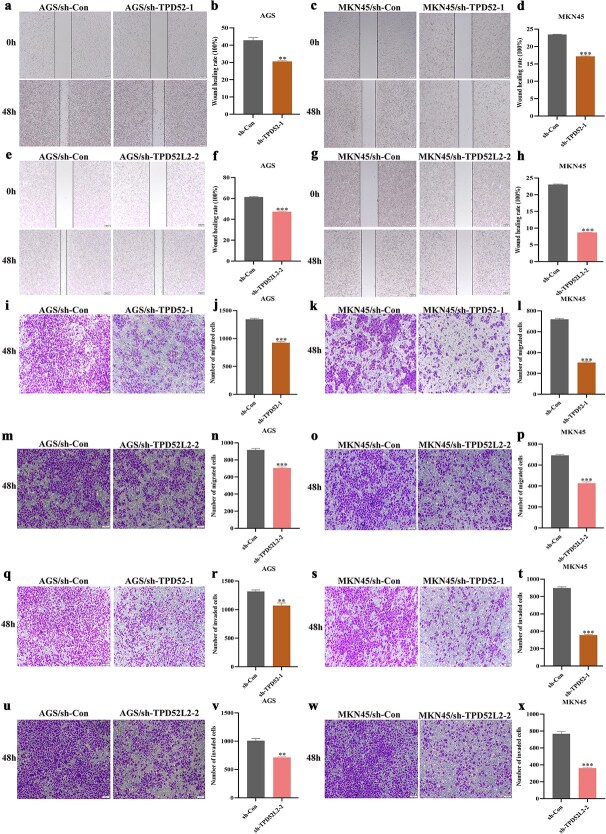
Influence of TPD52 and TPD52L2 knockdown on the migration and invasion of GC cells. (a–h) A wound healing assay was used to assess the impact of TPD52 and TPD52L2 knockdown on the lateral migration ability of GC cells. (i–p) Transwell assays were utilized to evaluate the influence of TPD52 and TPD52L2 knockdown on the longitudinal migration of GC cells. (q–x) Transwell assays were conducted to investigate the effects of TPD52 and TPD52L2 knockdown on the longitudinal invasion of GC cells (compared with the sh-Con groups, ^*^P < .05, ^**^P < .01, ^***^P < .001).

To assess the impact of TPD52 and TPD52L2 knockdown on GC cell motility and invasiveness, we performed wound healing and Transwell invasion assays. Compared with the sh-Con controls, both the sh-TPD52-1 and sh-TPD52L2-2 groups presented significantly lower wound closure rates (Fig. 11a–h), indicating impaired lateral migration capacity. Similarly, Transwell migration assays revealed that compared with control cells, MKN45 and AGS cells in the silenced groups (Fig. 11i–p) migrated at 48 h, confirming compromised longitudinal migration. To further support these findings, Transwell invasion assays with a Matrigel coating revealed markedly fewer membrane-traversing cells in the silenced groups than in the control groups (Fig. 11q–x), which was consistent with the suppressed ability of the Matrigel coating to degrade the extracellular matrix. Collectively, these data establish that TPD52 and TPD52L2 are critical regulators of both migratory and invasive phenotypes in GC cells.

### Knockdown of TPD52 and TPD52L2 induces cell cycle arrest

**Figure 12:**
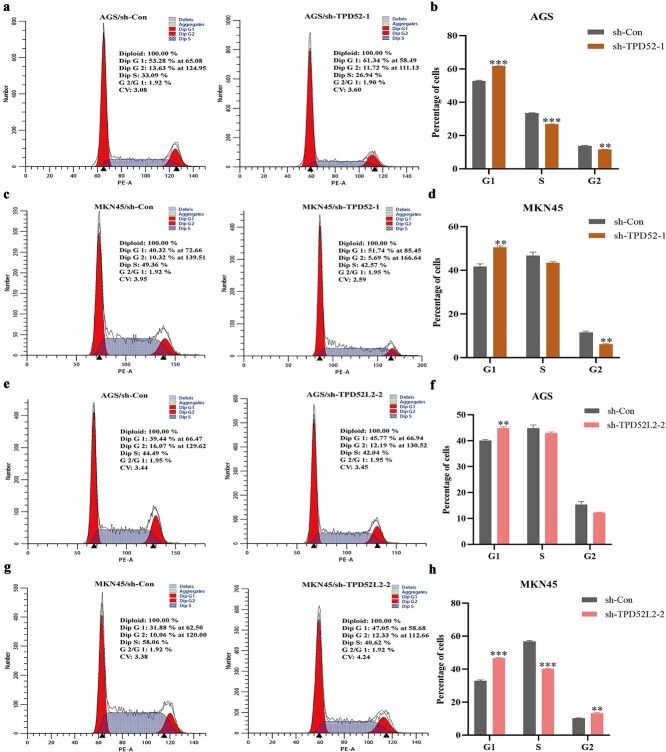
Cell cycle changes in GC cells after TPD52 and TPD52L2 were knocked down. (a–d) Effect of TPD52 knockdown on the cell cycle in GC. (e–h) Effect of TPD52L2 knockdown on the cell cycle in GC (compared with the sh-Con groups, ^*^P < .05, ^**^P < .01, ^***^P < .001).

Compared with the sh-Con group, the sh-TPD52-1 and sh-TPD52L2-2 groups presented a notable increase in the proportion of TPD52/TPD52L2-knockdown AGS and MKN45 cells in the G1 phase, whereas the percentage of cells in the S phase significantly decreased (Fig. 12a–h). These data demonstrate that TPD52/TPD52L2 knockdown induces G1/S checkpoint blockade, effectively arresting GC cell cycle progression at the G1 phase.​

### Knockdown of TPD52 and TPD52L2 promotes apoptosis

**Figure 13:**
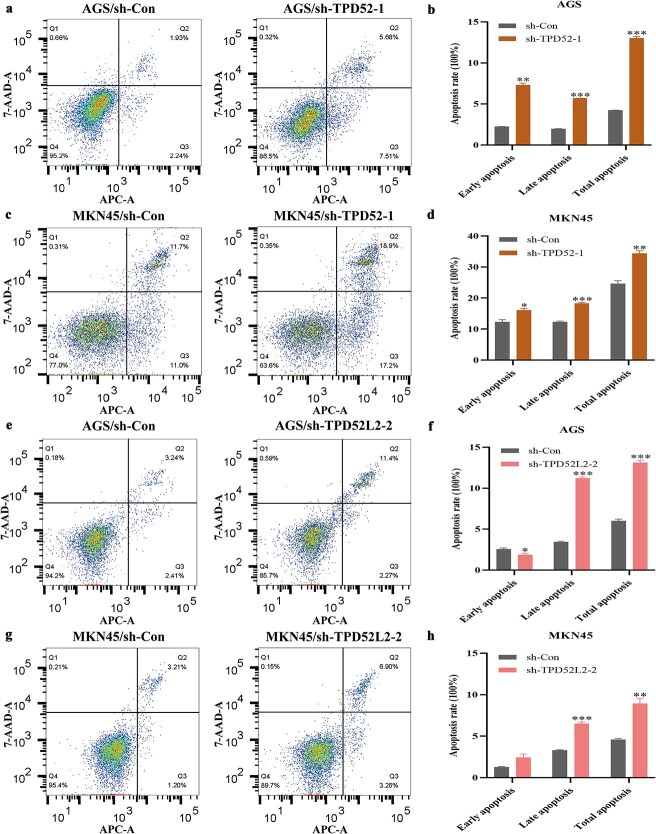
Apoptosis of GC cells after TPD52 and TPD52L2 were knocked down. (a–d) Effect of TPD52 knockdown on the apoptosis of GC cells. (e–h) Effect of TPD52L2 knockdown on the apoptosis of GC cells. (compared with the sh-Con groups, ^*^P < .05, ^**^P < .01, ^***^P < .001).

​Following TPD52/TPD52L2 knockdown in AGS and MKN45 GC cells, flow cytometry analysis with Annexin V/PI staining revealed significantly increased apoptosis.​​ Compared with the scramble shRNA controls (sh-Con), the sh-TPD52-1 and sh-TPD52L2-2 groups presented a significant increase​ in the total apoptosis rate of AGS cells and MKN45 cells. (Fig. 13a–h). In summary, these findings demonstrate that TPD52/TPD52L2 knockdown promotes intrinsic apoptosis *via* mitochondrial perturbation, suggesting their role as gatekeepers of GC cell survival.

### Effects of TPD52 and TPD52L2 knockdown on the protein expression phenotypes of gastric cancer cells

**Figure 14:**
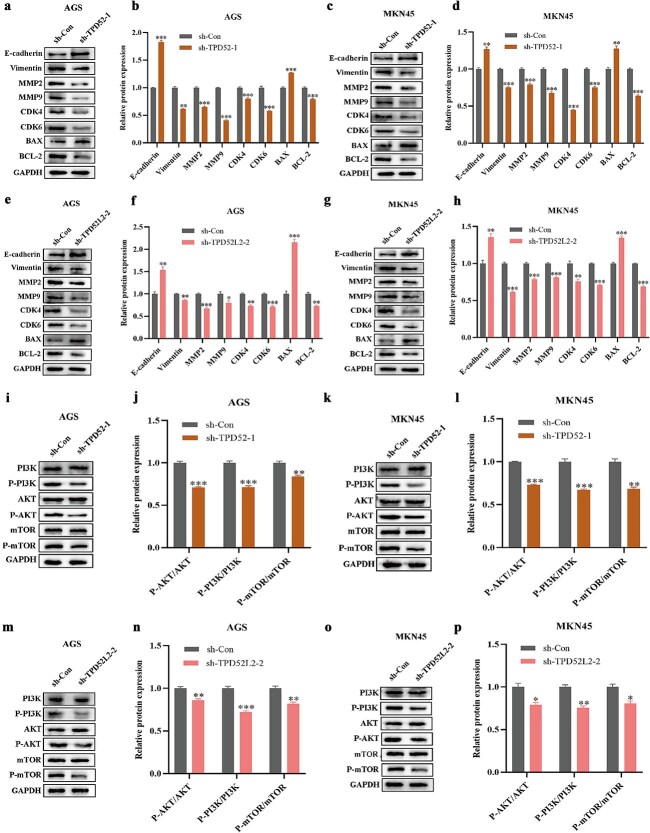
Effects of TPD52 and TPD52L2 knockdown on the expression of proteins related to migration and invasion, the cell cycle, apoptosis, and the PI3K/AKT/mTOR signaling pathway. (a–h) Effects of TPD52 and TPD52L2 knockdown on the protein expression of E-cadherin, vimentin, MMP2, MMP9, CDK4, CDK6, BAX, and BCL-2 in GC cells. (i–p) Effects of TPD52 and TPD52L2 knockdown on the protein expression of PI3K, P-PI3K, AKT, P-AKT, mTOR, and P-mTOR in GC cells. (compared with the sh-Con groups, ^*^P < .05, ^**^P < .01, ^***^P < .001).

Following TPD52/TPD52L2 knockdown, western blot analysis of cell cycle regulators revealed that CDK4/6 and BCL-2 levels were decreased, whereas BCL2 associated X (BAX) expression was increased in the sh-TPD52-1 and sh-TPD52L2-2 groups compared with the sh-Con controls (Fig. 14a–h). These findings suggest that TPD52/TPD52L2 depletion inhibits proliferation by inducing cell cycle arrest and apoptosis. Epithelial-mesenchymal transition (EMT) markers were examined to evaluate the impact of EMT on migration and invasion. TPD52/TPD52L2 suppression decreased the expression of the matrix metalloproteinases MMP2/MMP9 and the mesenchymal marker vimentin while increasing the expression of the epithelial marker E-cadherin (Fig. 14a–h). These findings suggest that TPD52/TPD52L2 knockdown attenuates GC cell motility by preventing EMT progression.

### Knockdown of TPD52 and TPD52L2 inhibited the PI3K/AKT/mTOR signaling pathway

The activation of the PI3K/AKT/mTOR pathway, which controls important cellular functions, encourages cancer by promoting unchecked angiogenesis, invasion, and proliferation. We examined changes in protein expression upon gene knockdown to determine whether TPD52/TPD52L2 affects the progression of GC through this route. TPD52/TPD52L2 knockdown dramatically decreased PI3K, AKT, and mTOR phosphorylation levels, as determined by western blot analysis (Fig. 14i–p). These results indicate that TPD52/TPD52L2 influences tumor cell survival and motility by activating the PI3K/AKT/mTOR pathway, which promotes the progression of gastric cancer by influencing tumor cell survival and motility.

## Discussion

The D52 gene family of tumor proteins plays crucial roles in tumorigenesis and cancer progression. We therefore investigated the mechanistic and clinical relevance of TPD52/TPD52L2 in gastric carcinogenesis through multiomics analysis (TCGA/GEO databases, tissue microarrays) and functional validation. Both genes showed tumor-specific overexpression, which was correlated with advanced clinicopathological features and poor prognosis. Systematic characterization revealed their involvement in immune microenvironment remodeling, pathway activation (PI3K/AKT/mTOR), and chemotherapeutic resistance. Crucially, shRNA-mediated knockdown in GC cell lines has been shown to play essential roles in sustaining malignant proliferation, migration, and invasion, providing the first experimental evidence of their oncogenic signaling in GC progression.

TPD52 was initially found to be highly expressed in human breast cancer, located at chromosome 8q21 [11]—a region frequently amplified in tumors and closely associated with cell proliferation, apoptosis, and metastasis. In the present investigation, TPD52 exhibited tumor-specific overexpression in GC tissues, and its expression levels positively correlated with patient age and the lymph node metastasis burden. Functional enrichment revealed its association with apoptosis modulation, calcium-dependent cadherin-mediated intercellular adhesion (critical for tissue homeostasis), and activation of the p53/cell cycle signaling axis. *In vitro* knockdown of TPD52 suppressed GC cell proliferation, migration, and invasion while inducing G0/G1-phase arrest and apoptosis, which was mechanistically linked to its ability to regulate PI3K/AKT/mTOR pathway activity. Notably, dysregulation of the PI3K/AKT/mTOR pathway is well documented to drive diverse oncogenic processes in human tumors, including autophagy, apoptosis evasion, chemotherapy resistance, and metastasis, making it a key therapeutic target for GC treatment *via* pathway-specific inhibitors [38]. Collectively, our findings suggest that TPD52 drives gastric oncogenicity primarily through activating the PI3K/AKT/mTOR pathway, underscoring its dual role in driving malignant progression and mediating therapeutic resistance.

Prior studies established that TPD52 overexpression in pancreatic cancer drives tumor progression, where genetic suppression attenuated malignant phenotypes and xenograft growth through AKT inactivation [16]. Conversely, renal cell carcinoma shows paradoxical tumor-suppressive activity; TPD52 downregulation is correlated with disease progression, while its restoration inhibits proliferation, EMT, and xenograft growth through PI3K/AKT pathway modulation [39]. In colorectal cancer, elevated TPD52 expression independently predicts poor survival and is associated with advanced tumor stage, metastasis, and enhanced invasiveness *via Focal adhesion kinase (*FAK)/integrin and PI3K/AKT-mediated EMT activation, positioning it as a potential therapeutic target [40]. In addition, TPD52 is regulated by various microRNA (miRNA) species, such as miR-34a, miR-449, and miR-1323, which can inhibit the proliferation and metastasis of breast cancer cells by targeting TPD52, and is involved in tumor progression [24, 25, 41–43]. miR-218, miR-224, and miR-103a-3p inhibited the growth, invasion, and metastasis of prostate cancer cells and promoted apoptosis *via* the downregulation of TPD52 expression [44–46]. Notably, emerging evidence highlights crosstalk between TPD52 and other signaling axes: the androgen receptor, for example, can inhibit lung cancer cell invasion by regulating the circ-SLCO1B7/miR-139-5p/TPD52 signaling pathway, thereby increasing sensitivity to cisplatin and effectively curbing nonsmall cell lung cancer development [47].

In addition to the previously mentioned findings, additional insights have emerged from single-cell analysis, which revealed that TPD52 is predominantly localized in plasma cells, CD8+ T cells, and mucosal epithelia. Notably, comparative immune profiling demonstrated that TPD52-high tumors exhibited increased infiltration of CD4+ memory T cells and mast cells, accompanied by concomitant depletion of CD8+ T cells. Consistently, lower ESTIMATE immune/stromal scores in the TPD52-high subgroup suggested that suppressing TPD52 might enhance immunotherapeutic responsiveness by remodeling the TME. This immunomodulatory role aligns with the broader paradigm of targeting tumor-associated antigens for immunotherapy development. Concurrently, tumor immunotherapy—particularly vaccine therapy—has emerged as a focal point in tumor biology research. For example, TPD52 was identified as a potential tumor-associated antigen in breast cancer through immunoscreening of a cDNA expression library derived from breast cancer tissues [48]. Subsequently, active immunization of Bagg albino laboratory mouse (BALB/c) mice with mD52 and CpG/ODN induced tumor-specific cytotoxic T lymphocyte responses and generated anti-mD52 antibodies [49, 50]. At present, current evidence indicates that TPD52-targeted vaccines elicit antitumor immunity, positioning them as promising immunotherapeutic targets for cancer intervention.

The TPD52L2 gene is located at chromosome 20q13 [12] and is widely expressed in human tumors. In this study, TPD52L2 was highly expressed in GC tissues compared with normal tissues. Notably, females and those in the TP53 mutation, grade 1, and N0 groups presented higher TPD52L2 expression levels than did those in the other subgroups. Concurrently, Kaplan–Meier curves revealed that patients with high TPD52L2 expression had longer overall survival than those with low TPD52L2 expression. Additionally, the diagnostic ROC curve results indicated that TPD52L2 had favorable predictive efficacy for clinical outcomes. Functional enrichment analysis (GO and KEGG) revealed that TPD52-related genes were involved in pathways such as the DNA damage response, regulation of the cell cycle, activation of the innate immune response, mitotic G1 DNA damage checkpoint signaling, cadherin binding, P53 binding, viral carcinogenesis, and resistance to the EGFR tyrosine kinase inhibitor. Notably, the AUC values (TPD52: 0.813; TPD52L2: 0.807) were comparable to those of clinically used markers such as CEA, CA19-9, and CA72-4, although prospective validation is needed for clinical implementation.

Our functional studies identified TPD52L2 as a pancancer oncogene. In GC, its knockdown suppressed proliferation, migration, and invasion while inducing cell cycle arrest and apoptosis. Clinically, its prognostic relevance has been validated across multiple cancers: elevated TPD52L2 independently predicts biochemical recurrence in prostate cancer [51], is correlated with poor survival and immune dysregulation in renal clear cell carcinoma [52], and drives glioma progression through bypass of G0/G1 arrest [26, 53]. Mechanistically, TPD52L2 modulates integrin-mediated ECM adhesion in oral squamous carcinoma *via* talin1-dependent AKT activation [54].

In particular, TPD52L2 may be a marker of acute lymphoblastic leukemia, and TPD52 and TPD52L2 are frequently coexpressed in childhood leukemia [55]. TPD52L2 was predominantly enriched in pit/gland mucous cells. High-TPD52L2 tumors exhibit an immunosuppressive phenotype marked by increased numbers of resting NK cells and M0/M1 macrophages, along with reduced numbers of naive/memory B cells and CD8+ T cells. Consistently, low-TPD52L2 tumors presented increased immune/ESTIMATE scores and superior immunotherapy responsiveness (TCIA-validated elevated IPS scores). Chemosensitivity profiling revealed that low-TPD52L2 tumors were vulnerable to lapatinib, whereas high-TPD52L2 tumors were susceptible to docetaxel, doxorubicin, gemcitabine, rapamycin, and vinorelbine. Critically, TPD52L2 upregulation in oxaliplatin-resistant GC models drives chemoresistance, whereas TPD52L2 suppression reverses this phenotype, suggesting actionable targets to overcome multidrug resistance. Mechanistically, TPD52L2 knockdown triggered endoplasmic reticulum (ER) stress–mediated apoptosis in drug-resistant GC cells *via* upregulation of the PERK/GRP78/CHOP/IRE1α axis and PARP/caspase-3 cleavage [28]. Conversely, in breast cancer models, TPD52L2 suppression confers metformin resistance through Pyruvate Dehydrogenase (PDH) inhibition, attenuating mitochondrial oxidative phosphorylation and ROS generation, indicating its predictive value for the metformin response [56]. While both TPD52 and TPD52L2 drive PI3K/AKT activation, their immune-modulatory effects differ significantly. High TPD52 correlated with CD4+ T-cell and mast cell infiltration but suppressed CD8+ T cells (Fig. 5g), whereas TPD52L2 elevated M0/M1 macrophages and depleted B cells (Fig. 5h). Drug sensitivity profiling further distinguished their roles: TPD52-high tumors were resistant to vinorelbine but sensitive to docetaxel/rapamycin (Fig. 7a–f), whereas TPD52L2-high tumors exhibited broad sensitivity to gemcitabine, doxorubicin, and lapatinib (Fig. 7g–l). These findings suggest that TPD52L2 may be a broader therapeutic target for combination chemotherapy.

In conclusion, our integrated multiomics analyses and experimental validation establish TPD52/TPD52L2 as a GC-specific oncoprotein with moderate prognostic and clinicopathological correlations. Mechanistically, their overexpression drives immunosuppressive microenvironments characterized by CD8+ T-cell depletion and macrophage polarization, which are correlated with diminished immunotherapy responsiveness. Computational analyses suggest a role for TPD52/TPD52L2 in shaping an immunosuppressive TME. However, direct evidence linking their expression to the immune checkpoint blockade response is lacking. Future studies should correlate TPD52/TPD52L2 levels with anti-PD-1 treatment outcomes in GC cohorts. Preliminary functional analyses demonstrated that genetic suppression of these genes attenuated malignant phenotypes through PI3K/AKT pathway inhibition and apoptosis induction. Positioning TPD52/TPD52L2 as potential diagnostic biomarkers and therapeutic targets. While this study delineates their translational implications in GC management, further mechanistic elucidation of their tumor-immune crosstalk and preclinical validation of targeted therapies remain imperative. Importantly, our findings position TPD52/TPD52L2 as actionable biomarkers for risk stratification and therapeutic targeting, particularly when combined with PI3K/AKT inhibitors or immunotherapy. Limitations of this study include its retrospective design and lack of *in vivo* validation; thus, future prospective cohorts and xenograft models are needed to confirm these findings. Future studies should include orthotopic xenograft models in immunodeficient mice (e.g. NOD/SCID) injected with AGS/MKN45 cells. The efficacy of TPD52/TPD52L2 knockdown was validated *via* intratumoral shRNA delivery, with tumor growth monitored weekly *via* bioluminescence imaging. Metastatic potential can be assessed *via* tail vein injection and lung nodule quantification.

**Key Points**

- Overexpression & prognosis: TPD52/TPD52L2 are overexpressed in gastric cancer (GC) and are correlated with advanced TNM stages and poor survival. TPD52L2 is an independent prognostic biomarker.
- Functional mechanism: Knockdown suppresses GC progression by inhibiting proliferation/migration/invasion, inducing apoptosis/G0/G1 arrest *via* PI3K/AKT/mTOR inactivation.
- Immune evasion: High expression is linked to reduced immune infiltration (e.g. CD8^+^ T/natural killer (NK) cells) and an immunosuppressive microenvironment (↑Tregs/TAMs), reducing immunotherapy efficacy.
- Diagnostic value: A moderate receiver operating characteristic curve (area under the curve >0.8) supports their role as early detection and prognostic biomarkers.
- Therapeutic target: Targeting TPD52/TPD52L2 enhances the chemotherapeutic (docetaxel/rapamycin) response and synergizes with immunotherapy, suggesting novel GC treatment strategies.

## Supplementary Material

Supplementary_table_1_4_elaf015

Supplementary_fig_1_elaf015

## Data Availability

All analyzed data are included in this published article. The original data are available upon reasonable request to the corresponding author.

## Declarations

### Availability of supporting data

The data supporting the results of this article are included in the article and its accompanying files.
