# Supplementary material for: Dual oncogenic roles of TPD52 and TPD52L2 in gastric cancer progression via PI3K/AKT activation and immunosuppressive microenvironment remodeling
Source: Brief Funct Genomics. 2025 Sep 19;24:elaf015. doi: 10.1093/bfgp/elaf015 (PMC12449195; doi:10.1093/bfgp/elaf015)
Supplement: Supplementary_table_1_4_elaf015 [file supplementary_table_1_4_elaf015.docx]

**Supplementary table 1 Univariate and multivariate Cox regression of clinicopathological features and different expression groups of TPD52 in TCGA dataset**

| Variables | Total(N) | Univariate Cox regression | | Multivariate Cox regression | |
| --- | --- | --- | --- | --- | --- |
|  |  | HR（95%CI） | P value | HR（95%CI） | P value |
| Gender | 370 |  |  |  |  |
| Male | 237 | Reference |  |  |  |
| Female | 133 | 0.789 (0.554 - 1.123) | 0.188 |  |  |
| Age | 367 |  |  |  |  |
| <= 65 | 163 | Reference |  | Reference |  |
| > 65 | 204 | 1.620 (1.154 - 2.276) | **0.005** | 1.815 (1.262 - 2.610) | **0.001** |
| Pathologic T stage | 362 |  |  |  |  |
| T1 | 18 | Reference |  | Reference |  |
| T2 | 78 | 6.725 (0.913 - 49.524) | 0.061 | 5.291 (0.711 - 39.368) | 0.104 |
| T3 | 167 | 9.548 (1.326 - 68.748) | **0.025** | 6.219 (0.851 - 45.464) | 0.072 |
| T4 | 99 | 9.634 (1.323 - 70.151) | **0.025** | 5.572 (0.748 - 41.529) | 0.094 |
| Pathologic N stage | 352 |  |  |  |  |
| N0 | 107 | Reference |  | Reference |  |
| N1 | 97 | 1.629 (1.001 - 2.649) | **0.049** | 1.374 (0.814 - 2.317) | 0.234 |
| N2 | 74 | 1.655 (0.979 - 2.797) | 0.060 | 1.471 (0.854 - 2.535) | 0.164 |
| N3 | 74 | 2.709 (1.669 - 4.396) | **< 0.001** | 2.363 (1.403 - 3.981) | **0.001** |
| Pathologic M stage | 352 |  |  |  |  |
| M0 | 327 | Reference |  | Reference |  |
| M1 | 25 | 2.254 (1.295 - 3.924) | **0.004** | 2.386 (1.310 - 4.344) | **0.004** |
| TPD52 | 370 |  |  |  |  |
| Low | 186 | Reference |  |  |  |
| High | 184 | 1.109 (0.798 - 1.541) | 0.539 |  |  |

**Supplementary table 2 Univariate and multivariate Cox regression of clinicopathological features and different expression groups of TPD52L2 in TCGA dataset**

| Variables | Total(N) | Univariate Cox regression | | Multivariate Cox regression | |
| --- | --- | --- | --- | --- | --- |
|  |  | HR（95%CI） | P value | HR（95%CI） | P value |
| Gender | 370 |  |  |  |  |
| Male | 237 | Reference |  |  |  |
| Female | 133 | 0.789 (0.554 - 1.123) | 0.188 |  |  |
| Age | 367 |  |  |  |  |
| <= 65 | 163 | Reference |  | Reference |  |
| > 65 | 204 | 1.620 (1.154 - 2.276) | **0.005** | 1.815 (1.262 - 2.610) | **0.001** |
| Pathologic T stage | 362 |  |  |  |  |
| T1 | 18 | Reference |  | Reference |  |
| T2 | 78 | 6.725 (0.913 - 49.524) | 0.061 | 5.291 (0.711 - 39.368) | 0.104 |
| T3 | 167 | 9.548 (1.326 - 68.748) | **0.025** | 6.219 (0.851 - 45.464) | 0.072 |
| T4 | 99 | 9.634 (1.323 - 70.151) | **0.025** | 5.572 (0.748 - 41.529) | 0.094 |
| Pathologic N stage | 352 |  |  |  |  |
| N0 | 107 | Reference |  | Reference |  |
| N1 | 97 | 1.629 (1.001 - 2.649) | **0.049** | 1.374 (0.814 - 2.317) | 0.234 |
| N2 | 74 | 1.655 (0.979 - 2.797) | 0.060 | 1.471 (0.854 - 2.535) | 0.164 |
| N3 | 74 | 2.709 (1.669 - 4.396) | **< 0.001** | 2.363 (1.403 - 3.981) | **0.001** |
| Pathologic M stage | 352 |  |  |  |  |
| M0 | 327 | Reference |  | Reference |  |
| M1 | 25 | 2.254 (1.295 - 3.924) | **0.004** | 2.386 (1.310 - 4.344) | **0.004** |
| TPD52L2 | 370 |  |  |  |  |
| Low | 184 | Reference |  |  |  |
| High | 186 | 0.980 (0.706 - 1.361) | 0.905 |  |  |

**Supplementary table 3 Univariate and multivariate Cox regression of clinicopathological features and different expression groups of TPD52 in GSE84437 dataset**

| Variables | Total(N) | Univariate Cox regression | | Multivariate Cox regression | |
| --- | --- | --- | --- | --- | --- |
|  |  | HR（95%CI） | P value | HR（95%CI） | P value |
| Gender | 433 |  |  |  |  |
| Male | 296 | Reference |  |  |  |
| Female | 137 | 0.796 (0.588 - 1.078) | 0.141 |  |  |
| Age | 433 |  |  |  |  |
| <= 65 | 283 | Reference |  | Reference |  |
| > 65 | 150 | 1.335 (1.012 - 1.761) | **0.041** | 1.327 (1.003 - 1.755) | **0.048** |
| Pathologic T stage | 433 |  |  |  |  |
| T1 | 11 | Reference |  | Reference |  |
| T2 | 38 | 0.948 (0.197 - 4.564) | 0.947 | 0.705 (0.144 - 3.446) | 0.666 |
| T3 | 92 | 2.583 (0.623 - 10.709) | 0.191 | 1.689 (0.400 - 7.132) | 0.476 |
| T4 | 292 | 3.965 (0.983 - 15.993) | 0.053 | 2.391 (0.580 - 9.848) | 0.228 |
| Pathologic N stage | 433 |  |  |  |  |
| N0 | 80 | Reference |  | Reference |  |
| N1 | 188 | 1.466 (0.940 - 2.285) | 0.091 | 1.449 (0.926 - 2.269) | 0.105 |
| N2 | 132 | 2.921 (1.876 - 4.548) | **< 0.001** | 2.553 (1.627 - 4.004) | **< 0.001** |
| N3 | 33 | 3.920 (2.229 - 6.894) | **< 0.001** | 3.090 (1.741 - 5.486) | **< 0.001** |
| TPD52 | 433 |  |  |  |  |
| Low | 216 | Reference |  |  |  |
| High | 217 | 0.973 (0.741 - 1.276) | 0.841 |  |  |

**Supplementary table 4 Univariate and multivariate Cox regression of clinicopathological features and different expression groups of TPD52L2 in GSE84437 dataset**

| Variables | Total(N) | Univariate Cox regression | | Multivariate Cox regression | |
| --- | --- | --- | --- | --- | --- |
|  |  | HR（95%CI） | P value | HR（95%CI） | P value |
| Gender | 433 |  |  |  |  |
| Male | 296 | Reference |  |  |  |
| Female | 137 | 0.796 (0.588 - 1.078) | 0.141 |  |  |
| Age | 433 |  |  |  |  |
| <= 65 | 283 | Reference |  | Reference |  |
| > 65 | 150 | 1.335 (1.012 - 1.761) | **0.041** | 1.327 (1.003 - 1.755) | **0.048** |
| Pathologic T stage | 433 |  |  |  |  |
| T1 | 11 | Reference |  | Reference |  |
| T2 | 38 | 0.948 (0.197 - 4.564) | 0.947 | 0.705 (0.144 - 3.446) | 0.666 |
| T3 | 92 | 2.583 (0.623 - 10.709) | 0.191 | 1.689 (0.400 - 7.132) | 0.476 |
| T4 | 292 | 3.965 (0.983 - 15.993) | 0.053 | 2.391 (0.580 - 9.848) | 0.228 |
| Pathologic N stage | 433 |  |  |  |  |
| N0 | 80 | Reference |  | Reference |  |
| N1 | 188 | 1.466 (0.940 - 2.285) | 0.091 | 1.449 (0.926 - 2.269) | 0.105 |
| N2 | 132 | 2.921 (1.876 - 4.548) | **< 0.001** | 2.553 (1.627 - 4.004) | **< 0.001** |
| N3 | 33 | 3.920 (2.229 - 6.894) | **< 0.001** | 3.090 (1.741 - 5.486) | **< 0.001** |
| TPD52L2 | 433 |  |  |  |  |
| Low | 216 | Reference |  |  |  |
| High | 217 | 1.224 (0.933 - 1.607) | 0.145 |  |  |
