# Supplementary figures and images for: Dual oncogenic roles of TPD52 and TPD52L2 in gastric cancer progression via PI3K/AKT activation and immunosuppressive microenvironment remodeling
Source: Brief Funct Genomics. 2025 Sep 19;24:elaf015. doi: 10.1093/bfgp/elaf015 (PMC12449195; doi:10.1093/bfgp/elaf015)

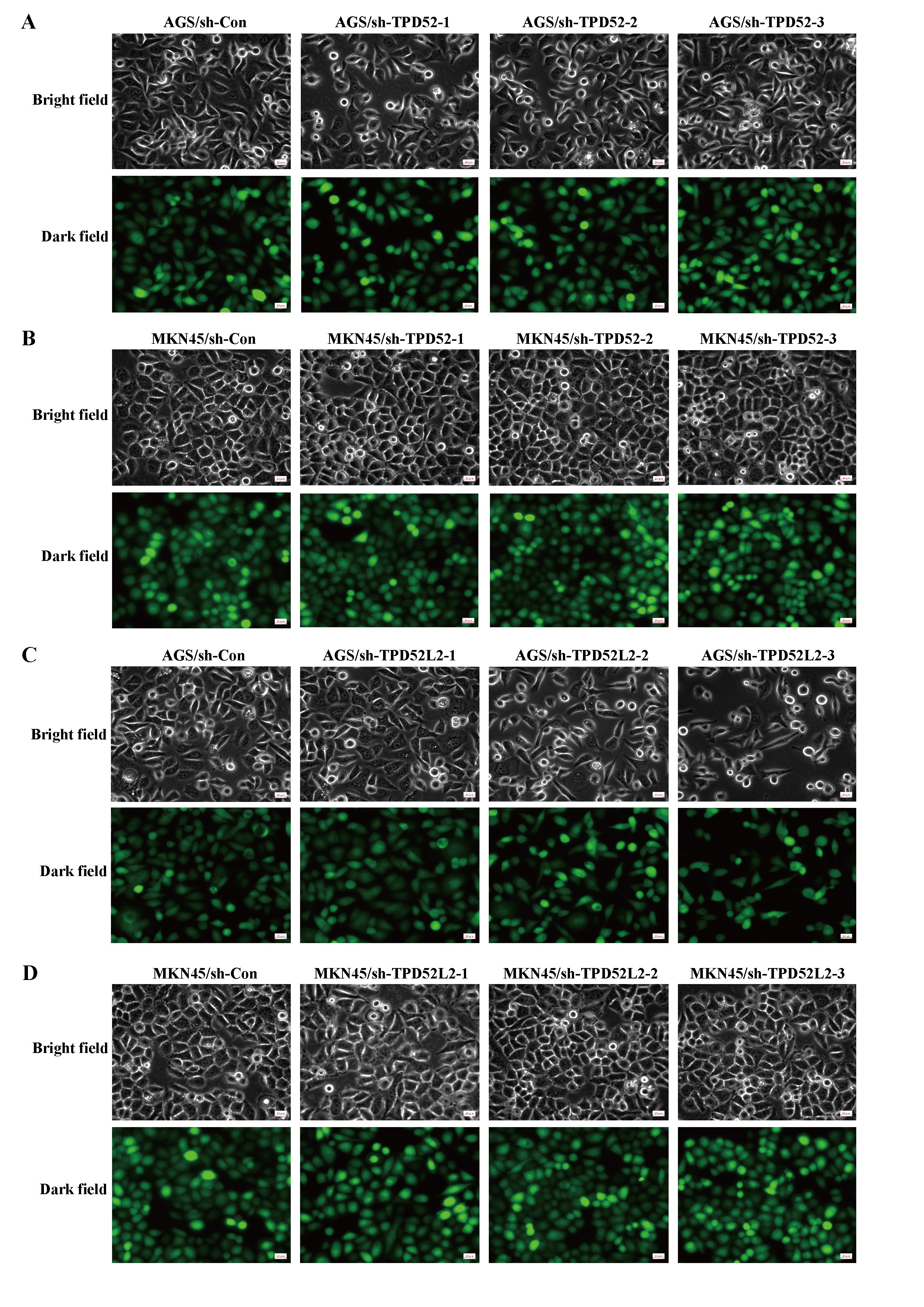

Supplement: Supplementary_fig_1_elaf015 [file supplementary_fig_1_elaf015.jpeg]
